# Quality of cardiovascular disease care in Ontario, Canada: missed opportunities for prevention - a cross sectional study

**DOI:** 10.1186/1471-2261-12-74

**Published:** 2012-09-12

**Authors:** Clare Liddy, Jatinderpreet Singh, William Hogg, Simone Dahrouge, Catherine Deri-Armstrong, Grant Russell, Monica Taljaard, Ayub Akbari, George Wells

**Affiliations:** 1 C.T. Lamont Primary Health Care Research Centre, Bruyère Research Institute, Ottawa, ON, Canada; 2 Department of Family Medicine, University of Ottawa, Ottawa, ON, Canada; 3 Institute of Population Health, University of Ottawa, Ottawa, ON, Canada; 4 Department of Economics, University of Ottawa, Ottawa, ON, Canada; 5 Southern Academic Primary Care Research Unit, Victoria, Australia; 6 School of Primary Health Care, Monash University, Victoria, Australia; 7 Clinical Epidemiology Program, Ottawa Hospital Research Institute, Ottawa, ON, Canada; 8 Department of Epidemiology and Community Medicine, University of Ottawa, Ottawa, ON, Canada; 9 Department of Medicine, Division of Nephrology, University of Ottawa, Ottawa, ON, Canada; 10 Cardiovascular Research Methods Centre, Ottawa Heart Institute, Ottawa, ON, Canada

**Keywords:** Cardiovascular disease, Primary care, Diabetes, Evidence-based care, Preventive care, Quality of care

## Abstract

**Background:**

Primary care plays a key role in the prevention and management of cardiovascular disease (CVD). We examined primary care practice adherence to recommended care guidelines associated with the prevention and management of CVD for high risk patients.

**Methods:**

We conducted a secondary analysis of cross-sectional baseline data collected from 84 primary care practices participating in a large quality improvement initiative in Eastern Ontario from 2008 to 2010. We collected medical chart data from 4,931 patients who either had, or were at high risk of developing CVD to study adherence rates to recommended guidelines for CVD care and to examine the proportion of patients at target for clinical markers such as blood pressure, lipid levels and hemoglobin A1c.

**Results:**

Adherence to preventive care recommendations was poor. Less than 10% of high risk patients received a waistline measurement, half of the smokers received cessation advice, and 7.7% were referred to a smoking cessation program. Gaps in care exist for diabetes and kidney disease as 54.9% of patients with diabetes received recommended hemoglobin-A1c screenings, and only 55.8% received an albumin excretion test. Adherence rates to recommended guidelines for coronary artery disease, hypertension, and dyslipidemia were high (>75%); however <50% of patients were at target for blood pressure or LDL-cholesterol levels (37.1% and 49.7% respectively), and only 59.3% of patients with diabetes were at target for hemoglobin-A1c.

**Conclusions:**

There remain significant opportunities for primary care providers to engage high risk patients in prevention activities such as weight management and smoking cessation. Despite high adherence rates for hypertension, dyslipidemia, and coronary artery disease, a significant proportion of patients failed to meet treatment targets, highlighting the complexity of caring for people with multiple chronic conditions.

**Trial Registration:**

NCT00574808

## Background

Cardiovascular disease (CVD) is the leading cause of death and disability in Canada [1,2], with 447,000 hospitalizations and 3.9 million patient days in the hospital each year, contributing to an overall yearly cost of $18.5 billion [3-5]. With an increasingly aging Canadian population, the number of deaths and overall burden of CVD is predicted to double by the year 2018 [6].

Primary care providers are well placed to play a central role in preventing and managing CVD and other chronic diseases, as 95% of Canadian adults with chronic conditions in Canada report having a regular family physician [7]. The frequent visits these patients make to their primary care provider offer a unique opportunity to monitor patients’ cardiovascular health, encourage a healthy lifestyle, and ensure preventive care [8,9].

As part of a large quality improvement initiative based in 84 primary care practices in Eastern Ontario, Canada, we studied practice-level adherence to recommended guidelines, in nine areas of care including coronary artery disease, peripheral vascular disease, stroke/transient ischemic attack, chronic kidney disease, diabetes, dyslipidemia, hypertension, smoking cessation care, and weight management. To the best of our knowledge, this is the most comprehensive evaluation of CVD care to ever be conducted in primary care practices in Canada.

## Methods

### Study design

This is a secondary analysis of pooled cross-sectional baseline data collected through a larger quality improvement initiative known as the Improved Delivery of Cardiovascular Care (IDOCC) through Outreach Facilitation project ( http://www.idocc.ca) [10]. IDOCC received ethical approval from the Ottawa Hospital Research Ethics Board.

The objective of IDOCC was to assist primary care providers in improving their delivery of evidence-based care for the secondary prevention of heart disease, stroke, peripheral vascular disease, renal disease, and diabetes. IDOCC used trained facilitators who work with health care providers for 24 months within their practices to help them incorporate elements of the Chronic Care Model into daily care routines [11]. The IDOCC intervention was evaluated using a cluster randomized controlled trial. The intervention was delivered to groups of practices in three distinct steps (26–30 practices per step), with each consecutive step beginning the program approximately one year apart (i.e., Step I: 2008, Step II: 2009, Step III: 2010). Intervention delivery was randomized at the regional level, with each step containing three regions, for a total of nine regions.

The first stage of the IDOCC intervention consisted of a chart audit intended to understand the strengths and gaps in care delivery within each practice in order to inform the components of the facilitation intervention. These baseline data are used in this secondary analysis to report on the quality of CVD care in primary care practices in Eastern Ontario.

### Study setting

The geographic setting for this study was the Champlain Local Health Integration Network (LHIN), which is one of 14 regional health districts in Ontario and encompasses Ottawa and its surrounding communities. The Champlain LHIN is a culturally diverse region with a population of 1.2 million people who have chronic disease burdens and patient health outcomes which are comparable to Ontario and the rest of Canada [12].

### Sample

All practices providing general primary care services and planning to remain in operation for the subsequent two years were eligible to participate, regardless of the practice’s team structure, rurality, group size, or provider remuneration structure (fee-for-service, capitation, or salary-based). Walk-in only clinics were excluded.

We approached all 434 eligible practices in the Champlain LHIN with the intent of recruiting a minimum of 27 practices per step, as per the sample size requirements of the IDOCC study [10]. In each step, recruitment continued until at least 30 practices were enrolled. In total, 93 practices were recruited: 30, 33, and 30 in Steps I, II, and III, respectively. All participating practices completed a practice characteristic survey highlighting details about their practice organizational structure, including primary care model type and number of patients. Physicians also provided consent to allow the study investigators to access information about their practices stored in health administrative databases stored at the Institute for Clinical Evaluative Sciences (ICES). Further details about the practice recruitment process are presented elsewhere [10].

Medical chart audits for patients with or at high risk of CVD were used to assess each practice’s adherence to evidence-based guidelines for CVD care. Eligible patients for the chart audit were those over 40 years of age who met at least one of the following criteria: 1. Cardiovascular disease including coronary artery disease, cerebrovascular disease (stroke and/or transient ischemic attack), or peripheral vascular disease; 2. Diabetes mellitus; 3. Chronic kidney disease; 4. at high risk for cardiovascular disease based on the presence of at least three of the following cardiovascular risk factors as defined by the Framingham Risk Score: age (male ≥ 45, female ≥ 55), smoker, hypertension, and dyslipidemia [13].

### Data collection

Six chart abstractors audited medical chart data across the following four areas: 1. Cardiovascular disease/risk factor screening, 2. Drug prescriptions relating to CVD, 3. Referral to external programs (e.g., referral to smoking cessation programs) and 4. Clinical measures (e.g., blood pressure, lipid profiles, etc.). We designed a chart abstraction manual that included information about making initial contact with the practice, instructions for ensuring patient confidentiality, information on assessing patient eligibility (including a list of synonyms for each condition), data entry instructions, and a copy of the chart abstraction form containing the data elements to be collected from each chart. A more detailed description of the application of chart audits and the methodology used can be found in separate publications [10,14]. Patient charts were randomly selected using established chart sampling protocols, including the ‘tape measure method’ for paper and mixed charting systems (i.e., paper and electronic records) and a random number generator for practices using only electronic records [14]. We aimed to abstract a minimum of 45 charts from each practice. Chart abstractors were blinded to primary and secondary outcomes of the IDOCC study.

To ensure the quality of the abstracted data across chart abstractors, a four-part quality implementation and monitoring process was established. The process involved: 1. Standardized protocol implementation, 2. Extensive data abstraction training, 3. Continuous re-abstraction and validation among abstractors to monitor the inter-rater reliability, and 4. Constant chart abstractor feedback and re-training [15]. The baseline inter-rater reliability kappa value was 0.91 and the overall percent agreement was 94.3%.

In addition, we assessed potential selection biases by linking to administrative databases stored at ICES in order to compare practice, physician and patient-level profiles between participating physicians and physicians from the Champlain region who opted not to participate in IDOCC. We also collected data for all physicians within the province of Ontario in order to gain insight into the generalizability of our findings.

### Outcome measures

The selection of care indicators was based on recommendations from a locally developed integrated CVD prevention and management guideline tailored to primary care: The Champlain Primary Care Cardiovascular Disease Prevention and Management Guideline [16]. This guideline was derived by an expert panel of family physicians and specialists (e.g., cardiologists, endocrinologists, etc.) who critically reviewed and harmonized current national and international guidelines for CVD and its risk factors using the AGREE methodology [17]. The guideline is updated on an annual basis and is available in English and French. The guideline can be viewed at http://www.idocc.ca.

Processes of care: We assessed whether recommended care manoeuvres were performed, recommended, or discussed during the one year preceding the abstraction date. For example, for patients with diabetes, we examined whether two hemoglobin A1c (HbA1c) tests were recommended, discussed or performed in the chart audit year. A list of all of the process of care manoeuvres examined is presented in Table 1. Patients with multiple conditions were assessed separately for manoeuvres associated with each individual condition.

**Table 1:** Process of care manoeuvres

**Area of care**	Process of care manoeuvres*
Coronary Artery Disease	2 Blood pressure measures
	Lipid profile
	Fasting glucose
	ACE Inhibitor, Angiotensin receptor blocker, beta blocker
	ASA
Peripheral Vascular Disease	2 Blood pressure measures
	Lipid profile
	Fasting glucose
	ACE inhibitor and/or Angiotensin receptor blocker
	Lipid lowering medication
	ASA
Stroke	2 Blood pressure measures
	Lipid profile
	Fasting blood glucose
	ASA
	*If stroke within past year*
	Echo cardiogram
	Carotid doppler
	CT head scan
	EKG
Diabetes	Two hemoglobin A1c tests
	Glycemic control medication
	2 Blood pressure measures
	Lipid profile
	Albumin-to-creatinine ratio (ACR)
	Estimated glomerular filtration rate (eGFR)
Chronic Kidney Disease	ACR
	2 Blood pressure measures
	Lipid profile
Dyslipidemia	Lipid profile
	Lipid lowering medication
Hypertension	Two blood pressure readings
	Anti-hypertensive medication
Smoking	Smoking cessation counselling
	Smoking cessation program
	Smoking cessation drug
Obesity	Waist circumference
	Dietician or weight loss program

ACE – Angiotensin converting enzyme, ACR - Albumin-to-creatinine ratio, ASA – Acetylsalicylic acid.

CT – Computed Tomography, eGFR - Estimated glomerular filtration rate, EKG – Electrocardiogram.

* assessed whether recommended care manoeuvres were performed, recommended, or discussed during the one year preceding the abstraction date.

Clinical test results were also assessed to determine the proportion of individuals within the recommended target range for each of the following clinical markers: blood pressure (<130/80 mmHg for patients with diabetes or CKD and <140/90 mmHg for everyone else), Low-density lipoprotein (LDL) (<2.0 mmol/L), glycemic level (HbA1c < 7.0%, Fasting Blood Glucose (FBG) < 7 mmol and > 4 mmol/L) and proteinuria (Albumin-to-creatinine (ACR) < 40).

### Data analysis

Practice and patient characteristics are described using frequencies and proportions or means and standard deviations. Process of care indicators and clinical test results are described as proportions with 95% confidence intervals, adjusted for clustering of patients within practices [18]. All analyses were conducted using a commercially available software package (SAS, Version 9.2, SAS Institute Inc) [19].

## Results

194 physicians in 93 practices consented to participate in IDOCC. Compared to non-participants (i.e., those physicians in the Champlain region who declined to participate in IDOCC) and all physicians in Ontario, IDOCC physicians were more likely to be female (IDOCC: 54.3%, IDOCC non-participants: 43.0%, Ontario: 36.5%), trained in Canada (IDOCC: 90%, IDOCC non-participants: 82.1%, Ontario: 76.7%), and to practice in a clinic that employed a capitated remuneration model (IDOCC: 29.5%, IDOCC non-participants: 12.6%, Ontario: 16.5%).

Of the 93 practices that agreed to participate in IDOCC, nine practices dropped out prior to baseline data collection for various reasons including not having the office space to accommodate a chart abstractor, while several small practices did not have the 45 eligible patient charts necessary for entry into the study. The 84 participating practices varied in their team structure, physician remuneration approach, and rurality (Table 2).

**Table 2:** Practice Profile (n = 84)

**Characteristics**	**n (%)**
EMR	41 (48.8%)
Practice structure	
Solo physician	29 (34.5%)
Group single disciplinary	31 (36.9%)
Multidisciplinary	24 (28.6%)
Physician remuneration*	
FFS	43 (52.4%)
Capitation	27 (32.9%)
Salary- Community Health Centres	12 (14.6%)
Urban practices	69 (82.1%)

EMR – Electronic medical record, FFS – Fee-for-service.

* Two Long-term care centres were not included in this breakdown.

We collected medical chart data from an average of 59 patients per practice, for a total of 4,931 patients. Demographics and disease conditions for this group of patients are summarized in Table 3.

**Table 3:** Patient Profile (n = 4,931)

**Characteristics**	
Age (mean, SD)	66.4 (11.8)
Male (n,%)	2386 (48.4%)
Coronary Artery Disease (n,%)	1510 (30.6%)
Peripheral Vascular Disease (n,%)	318 (6.5%)
Stroke/Transient Ischemic Attack (n,%)	636 (12.9%)
Diabetes (n,%)	2308 (46.8%)
Chronic Kidney Disease (n,%)	916 (18.6%)
Hypertension (n,%)	3793 (76.9%)
Dyslipidemia (n,%)	4111 (83.4%)
Smoke (n,%)	1049 (21.2%)
# of Cardiovascular-related comorbidities (mean, SD)	2.8 (1.1)

### Adherence to processes of care delivery

Practices had a high level of adherence to recommended guidelines associated with coronary artery disease, hypertension and dyslipidemia, with adherence rates of 75% or higher for each manoeuvre associated with these conditions (Table 4).

**Table 4:** Adherence to guidelines across 84 primary car practices*

**Process of care manoeuvre**	**Percentage of patients receiving care [95% CI]**
**All** (n = 4931)	
2 Blood pressure measures	74.8% [71.3-78.3%]
Lipid profile	77.7% [74.9-80.4%]
Waist circumference measure	9.9% [6.7-13.0%]
Dietician/weight loss program referral	18.2% [14.3-22.2%]
Smoking status recorded	95.4% [93.9-97.0%]
**Coronary Artery Disease** (n = 1510)	
Fasting blood glucose	80.0% [77.2-82.8%]
Medication (ACE, Angiotensin receptor blocker, beta blocker)	88.5% [86.5-90.4%]
ASA	76.0% [73.2-78.9%]
**Peripheral Vascular Disease** (n = 318)	
Fasting blood glucose	78.9% [74.6-83.2%]
ACE inhibitor and/or Angiotensin receptor blocker	66.9% [61.4-72.5%]
Lipid lowering medication	83.3% [77.8-88.8%]
ASA	75.8% [70.3-81.2%]
**Stroke** (n = 636)	
Fasting blood glucose	76.9% [72.8-81.0%]
ASA	78.9% [74.7-83.2%]
*If stroke within past year (n = 71)*	
Echo cardiogram	47.9% [38.6-65.6%]
Carotid doppler	59.6% [43.7-74.7%]
CT head scan	66.2% [52.6-79.8%]
EKG	52.1% [38.6-65.6%]
**Diabetes** (n = 2308)	
Two hemoglobin A1c tests	54.9% [50.1-59.6%]
Glycemic control medication	80.5% [78.0-83.0%]
ACR	55.8% [50.6-61.0%]
eGFR	83.8% [80.8-86.8%]
**Chronic Kidney Disease** (n = 916)	
ACR	51.6% [46.3-57.0%]
**Dyslipidemia** (n = 4111)	
Lipid profile	82.8% [80.9-84.7%]
Lipid lowering medication	91.5% [90.1-92.8%]
**Hypertension** (n = 3793)	
Two blood pressure readings	79.4% [76.0-82.8%]
Anti-Hypertensive medication	94.2% [93.1-95.3%]
**Smoking** (n = 1049)	
Smoking cessation counselling	52.8% [46.9-58.8%]
Smoking cessation program	7.7% [5.1-10.6%]
Smoking cessation drug	23.1% [19.2-27.1%]

ACE Angiotensin converting enzyme, ACR Albumin to creatinine ratio, ASA Acetylsalicylic acid, CT Computed Tomography, eGFR estimated glomerular filtration rate, EKG Electrocardiogram.

* assessed whether recommended manoeuvres were performed, recommended, or discussed during the one year preceding the abstraction date.

Gaps in care delivery were seen for diabetes management as only about half (54.9%, 95% CI [50.1-59.6%]) of the patient group had two HbA1c measures done in one year. Similarly, measurement of albumin excretion (ACR) was also only done for approximately half (55.8%, 95% CI [50.6 - 61.0%]) of the patients with diabetes. ACR screening was also poor amongst patients with known chronic kidney disease (51.6%, CI [46.3-57.0%]).

There was a low level of adherence to preventive care guidelines. Waist circumference measurements were done in only 9.9% (95% CI [6.7 – 13.0%]) of these high risk patients, while few were referred for dietary advice (18.2%, 95% CI [14.3-22.2%]). The highest level of adherence for waist circumference measurement and referral for dietary advice was seen for patients with diabetes (14.3% [9.4-19.2%] and 25% [20.1-29.9%] respectively), however, levels were still quite low. Similarly, less than half of the smokers had documented evidence of receiving smoking cessation advice (52.8%, 95% CI [46.9-58.8%]), 7.7% (95% CI [5.1-10.6%]) were referred to a smoking cessation program, and less than a quarter (23.1%, 95% CI [19.2-27.1%]) were recommended or prescribed any pharmacotherapy to assist with quitting.

### Patient clinical measures

Less than half of all high risk patients who had a lipid profile or blood pressure measure (37.1%, 95% CI [34.3-39.8%]; and 49.7%, 95% CI [47.1-52.3%] respectively) were within the recommended target range (Table 5). Patients that did not have established CVD, diabetes, or chronic kidney disease, but were at high risk for CVD based on the presence at least three risk factors, had the poorest control of LDL levels, while the diabetes patient sub-group had the poorest control of their blood pressure levels. Furthermore, only 59.3% (95% CI [56.6-62.0%]) of patients with diabetes met the clinical target for HbA1c levels (<7.0%).

**Table 5:** Percentage of patients at clinical targets

**Clinical Outcome**	Percentage of Patients at Target* Levels [95% CI]
**All**	
Blood pressure (n = 4581)	49.7% [47.1-52.3%]
LDL (n = 3661)	37.1% [34.3-39.8%]
Fasting blood glucose (n = 3892)	70.5% [68.5-72.5%]
ACR (n = 1534)	95.8% [94.8-96.7%]
**Coronary Artery Disease**	
Blood pressure (n = 1393)	60.2% [56.3-64.1%]
LDL (n = 1094)	51.7% [47.5-56.0%]
Fasting blood glucose (n = 1190)	77.1% [74.6-79.5%]
**Peripheral Vascular Disease**	
Blood pressure (n = 302)	54.0% [47.6-60.3%]
LDL (n = 219)	52.5% [44.8-60.3%]
Fasting blood glucose (n = 247)	73.7% [69.2-78.2%]
**Stroke**	
Blood pressure (n = 588)	59.9% [55.0-64.7%]
LDL (n = 403)	44.4% [39.4-49.4%]
Fasting blood glucose (n = 475)	79.4% [76.0-82.8%]
**Diabetes**	
Hemoglobin A1c (n = 1924)	59.3% [56.6-62.0%]
Blood pressure (n = 2137)	34.1% [31.1-37.2%]
LDL (n = 1793)	47.1% [43.4-50.9%]
ACR (n = 1155)	95.3% [94.2-96.4%]
**Chronic Kidney Disease**	
ACR (n = 435)	89.0% [86.4-91.5%]
Blood pressure (n = 867)	40.6% [35.8-45.4%]
LDL (n = 660)	48.8% [44.2-53.4%]
**Dyslipidemia**	
LDL (n = 3262)	39.7% [36.9-42.5%]
**Hypertension**	
Blood pressure (n = 3587)	46.9% [44.1-49.7%]
**Risk Factor Only**^†^	
Blood pressure (n = 1042)	62.8% [58.6-66.9%]
LDL (n = 835)	13.8% [11.3-16.3%]
Fasting blood glucose (n = 839)	97.3% [96.1-98.4%]

ACR – Albumin to creatinine ratio, LDL – Low density lipoprotein.

* **Target levels**:

Blood pressure: <130/80 for those patients that have diabetes and/or chronic kidney disease and 140/90 for all other patients.

LDL < 2.0 mmol/L, ACR < 40, Fasting Blood Glucose (<7 mmol/L, > 4 mmol/L), HbA1c < 7.0%.

† Patients that did not have established cardiovascular disease, diabetes or chronic kidney disease, but were at high risk for cardiovascular disease based on the presence of at least three of the following cardiovascular risk factors: age (males ≥ 45, females ≥ 55), smoker, hypertension, and dyslipidemia.

## Discussion

We found significant gaps in recommended care for this high risk CVD patient group, particularly for prevention activities such as smoking cessation and weight management. A reduction in the smoking rate of high risk CVD patients could significantly impact mortality [20-22]. Despite the existence of community help programs and public health campaigns, we found low rates for counseling patients to quit smoking, smoking cessation medication prescribing, and referrals to community programs, findings that are consistent with studies conducted across Canada, Europe and the United States [23-27]. This is a missed opportunity for prevention as primary care physicians are seen as a credible source of information, and a number of studies have shown that counselling in combination with a nicotine replacement therapy can double ones chances of successfully quitting [28]. Lack of time, competing demands, lack of reimbursement, and perceived patient resistance are barriers to care [24]. Improvements in organizing care at the level of the practice may reduce these barriers [24], along with patient self-management approaches such as motivational counselling [29], as well as greater linkages between the primary care practice and the community.

Our findings also highlight the significant ongoing gap in diabetes management within primary care practices in Eastern Ontario. Optimizing care and achieving clinical targets could reduce mortality as people with diabetes are at high risk of suffering a cardiovascular event [30-33]. These findings are consistent with other international findings, as a number of studies have demonstrated the challenges associated with treating patients with diabetes to target levels [34-36].

Furthermore, the results of this study are consistent with other findings in Canada and Ontario which have demonstrated high levels of adherence to guidelines associated with hypertension [37-39]. The adherence rates seen in this and other Canadian studies have been higher than those in the United States and other developed nations [40,41]. This marked improvement in hypertension management is likely due in part to local community programs, greater awareness and the establishment of the Canadian Hypertension Education Program (CHEP), an initiative developed in the late nineties to support primary care providers and patients by providing them with guidelines and recommendations for managing and preventing hypertension [42].

We found a spectrum in uptake patterns for new evidence in primary care. For example, although the benefits of measuring eGFR levels in diagnosing and managing chronic kidney disease were not published until 2004 [43], uptake of this guideline in primary care has been rapid at least in part to the automatic reporting of eGFR by laboratories when serum creatinine is requested. This is apparent as 84% of patients with diabetes had undergone eGFR testing in this study. Similar uptake patterns have been seen internationally as well [44,45]. In contrast, despite evidence that waistline is a strong predictor of cardiovascular related morbidity and mortality and has been recommended since 1998 [46], only one in ten patients had a measure done [47,48]. Low levels of uptake have also been documented in other countries [49,50]. One study reported that family physicians cited lack of time, extra workload, opportunity costs, and concerns about the acceptability of this manoeuvre as barriers to uptake [50].

Despite high adherence by providers to recommended guidelines for patients with hypertension, dyslipidemia and coronary artery disease, a high proportion of patients still did not meet clinical target levels, an observation that is likely due to several factors. For example, low patient compliance to medications could have impacted poor control rates, however, we are unable to confirm this from chart audit data alone. Also, changing guideline targets such as the recent changes made to the Canadian Lipid Guidelines in 2006, which decreased the target LDL level from 2.5 mmol/L to the more stringent 2.0 mmol/L may have also impacted the rates [51]. Furthermore, some experts have suggested that the choice of medication and a lack of dose titration are two potential reasons for poor LDL control rates [52], while a recent study suggested that 38% of high risk patients would be unable to reach an LDL target of <2.0 mmol/L even when using a maximum dose statin monotherapy [53]. As well, this difficulty in management could potentially be due in part to clinical inertia - resistance of a health care provider to intensify therapy when indicated – as there could be a need to change drug therapies, doses or initiate counselling to change lifestyle habits (e.g., diet, exercise, etc.). Alternately, the poor control rates may simply highlight the complexities of controlling LDL and blood pressure levels in high risk multimorbid patients.

Translating research evidence into practice is challenging as the research which underpins clinical guidelines is often obtained from studies that exclude patients with multimorbidities like the ones examined in this study [54-56]. As such, a cogent case can be made that guidelines may not easily apply to patients with multimorbidities. For example, if a multimorbid patient is newly diagnosed with hypertension and is already taking multiple medications, it may be less appropriate for a primary care physician to prescribe an additional drug. This is a concern when interpreting papers that look at guideline adherence, as most simply report on whether a treatment was delivered or not, even when it may be inappropriate to do so. In our study, as we wanted to capture whether appropriate care was delivered, we recorded physicians as adhering to a guideline if the specific care manoeuvre was performed, recommended or considered, regardless of whether the patient followed the recommendation such as compliance to prescriptions. This could also explain some of the gaps between processes and clinical outcomes in our results.

### Study limitations

Although the participants in this study represent diverse family practices, they all voluntarily participated in IDOCC resulting in a potential selection bias. We found differences in physician sex, training background and remuneration model between IDOCC participants and both non-participants and provincial averages. This bias may impact the generalizability of our findings, as practices that tend to take part in quality improvement initiatives such as IDOCC, are likely highly motivated and are higher performing than provincial averages. As such, we anticipate that the gaps in care observed in this study are likely greater amongst the entire practice population in Eastern Ontario. Also, we were only able to present clinical test results for those who had screenings during the chart audit year, and thus, we are unable to comment on control rates for those who did not have any tests done. We presented summary indicators of the quality of CVD care using baseline data from three groups of primary care practices enrolled over three distinct steps. Although these data were collected over a three year period (2007–2009), we did not explore the presence of any trends in quality over time, because trends would have likely been confounded with observed differences among the groups of practices allocated to the different steps in the stepped wedge design [57]. Lastly, this study had a measurement bias. As with all studies relying on chart audits, we could only assess guideline adherence through implication - activities performed but not charted would not have been captured by our methodology. This is less of a problem when adherence is measured by the performance and interpretation of a pathology test, than when dependent on documentation of clinical activities (such as providing advice to smokers to quit). Another limitation of using medical chart data was that we were unable to determine whether patients actually complied with medical drug prescriptions that were given to them by their provider, a factor that may have played a role in patients being unable to reach clinical targets. Notwithstanding the above limitations, chart audits are a rich source of information and remain the standard for capturing process of care data, as alternative methods of data collection at the practice are expensive and not feasible for large trials such as this.

## Conclusions

There remain significant opportunities for primary care providers to engage high risk CVD patients in prevention activities such as weight management and smoking cessation. In addition, despite high adherence rates for hypertension, dyslipidemia, and coronary artery disease, a significant proportion of patients failed to meet treatment targets highlighting the complexity of caring for people with multiple chronic conditions.

## Abbreviations

CVD: Cardiovascular Disease; FBG: Fasting Blood Glucose; HbA1c: Hemoglobin A1c; ICES: Institute for Clinical Evaluative Sciences; IDOCC: Improved Delivery of Cardiovascular Care; LDL: Low Density Lipoprotein; LHIN: Local Health Integration Network.

## Competing interests

The authors have no competing interests to declare.

## Authors’ contributions

CL and WH originally conceived and designed the study protocol. CDA and GR also contributed to the conception of this study. JS, MT and GW contributed to the analysis plan. All authors have contributed substantially to the ongoing project implementation and have participated in the preparation and review of this article. All authors read and approved the final manuscript.

## Pre-publication history

The pre-publication history for this paper can be accessed here:

http://www.biomedcentral.com/1471-2261/12/74/prepub
